# Histiocytic necrotizing lymphadenitis with hemophagocytic lymphohistiocytosis in adults: A single‐center analysis of 5 cases

**DOI:** 10.1002/iid3.1202

**Published:** 2024-02-27

**Authors:** Qingqing Chen, Jing Zhang, Huijun Huang, Tonglu Qiu, Ze Jin, Yu Shi, Huayuan Zhu, Lei Fan, Jianyong Li, Wenyu Shi, Yi Miao

**Affiliations:** ^1^ Department of Hematology, Pukou CLL Center The First Affiliated Hospital of Nanjing Medical University, Jiangsu Province Hospital, Collaborative Innovation Center for Cancer Personalized Medicine Nanjing China; ^2^ National Clinical Research Center for Hematologic Diseases The First Affiliated Hospital of Soochow University Suzhou China; ^3^ Department of Oncology Affiliated Hospital of Nantong University Nantong Jiangsu China; ^4^ Department of Hematology Affiliated Hospital of Nantong University Nantong Jiangsu China

**Keywords:** histiocytic necrotizing lymphadenitis, hemophagocytic lymphohistiocytosis, Kikuchi–Fujimoto disease

## Abstract

**Background:**

Histiocytic necrotizing lymphadenitis (HNL) is a self‐limited inflammatory disease of unknown pathogenesis. A very small fraction of patients with HNL could develop hemophagocytic lymphohistiocytosis (HLH), a hyperinflammatory disorder. These patients are diagnosed as HNL with HLH (HNL‐HLH). HNL‐HLH in the pediatric population has been systemically studied, however, the clinical, laboratory, and radiological features and outcomes of adult patients with HNL‐HLH remain to be explored. We aimed to explore the clinical, laboratory, and radiological features and outcomes of adult patients with HNL‐HLH.

**Methods:**

We collected the clinical data of patients with HNL‐HLH admitted to the First Affiliated Hospital of Nanjing Medical University from October 2010 to June 2015. All the patients underwent lymph node biopsy and have a pathological diagnosis of HNL. The age, gender, clinical presentation, lymph node signs, laboratory findings and imaging data, and pathological findings of the patients were collected.

**Results:**

In this study, we reported five adult patients with HNL‐HLH. All five patients showed enlarged lymph nodes and prolonged fever. Laboratory findings were consistent with the diagnosis of HLH. 18F‐fluorodeoxyglucose positron emission tomography/computed tomography (18F‐FDG PET/CT) showed enlarged lymph nodes with increased FDG uptake and splenic hypermetabolism could be present. All the patients responded well to corticosteroids and had a good prognosis. Two of the five patients were diagnosed with systemic lupus erythematosus during the follow‐up.

**Conclusions:**

Our study demonstrated that adult patients with HNL‐HLH showed distinct clinical, laboratory, and radiological features. And the prognosis is good and patients could be managed with steroids and supportive care.

## Graphical abstract

Our study demonstrated that adult patients with histiocytic necrotizing lymphadenitis‐hemophagocytic lymphohistiocytosis showed distinct clinical, laboratory, and radiological features. And the prognosis is good and patients could be managed with steroids and supportive care.

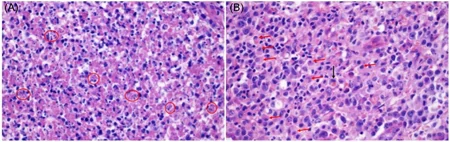

## 1 INTRODUCTION

Histiocytic necrotizing lymphadenitis (HNL) is a benign, self‐limiting disease that presents with few nonspecific symptoms such as fever and swollen lymph nodes in the neck. This disease was first described by Kikuchi and Fujimoto et al., it is also known as Kikuchi–Fujimoto disease (KFD) and often affects young women.^1^, ^2^ Until now, the mechanisms underlying HNL remain to be fully determined. Hemophagocytic lymphohistiocytosis (HLH) is a syndrome that is characterized by an excessive inflammatory response due to abnormal immune regulation. Patients usually present with persistent fever, pancytopenia, hepatosplenomegaly, increased ferritin, and bone marrow (BM) phagocytosis.^3^, ^4^ A small number of HNL patients can present with HLH which are called HNL‐HLH. HNL‐HLH patients show clinical features that are typical of HLH, including fever, hepatosplenomegaly, and cytopenia. However, HNL‐HLH patients also show some distinct clinical features as compared with other patients with HLH, including a high prevalence of central nervous system (CNS) symptoms and skin rash.^5^ Most cases with HNL‐HLH in the literature were pediatric cases. Most of pediatric patients could be managed with corticosteroids and supportive care and only a small minority of patients were treated with chemotherapy.^5^ Most patients had a good prognosis and the fatality rate was less than 10%.^5^ Until now, the clinical features and prognosis of HNL‐HLH in adults remain to be determined. Only 16 cases were described in case reports^6^, ^7^, ^8^, ^9^, ^10^, ^11^, ^12^, ^13^, ^14^, ^15^, ^16^, ^17^, ^18^, ^19^, ^20^, ^21^ and no case series were reported. In the study, we retrospectively analyzed the clinical features, laboratory, imaging, and pathological data, treatments, and outcomes of patients with HNL‐HLH.

## 2 MATERIALS AND METHODS

### 2.1 Study population

This study was a single‐center retrospective study. We included patients who presented at the First Affiliated Hospital of Nanjing Medical University. The inclusion criteria were as follows: (1) age ≥18 years; (2) histopathological diagnosis of HNL; (3) HLH developed during the course of HNL and HLH was not attributed to other causes. The diagnosis of HLH was established according to the HLH‐2004 criteria. Cases of HLH should fulfill five or more of the following criteria: (1) temperature ≥38.5°C; (2) splenomegaly; (3) cytopenia affecting at least two of three lineages in the peripheral blood: hemoglobin < 90 g/L, platelets < 100 × 10^9^/L or neutrophils < 1 × 10^9^/L; (4) hypertriglyceridemia and/or hypofibrinogenemia: triacylglycerol ≥ 3 mmol/L, or fibrinogen ≤ 1.5 g/L; (5) serum ferritin (SF) ≥ 500 µg/L; (6) hemophagocytosis present in BM or spleen or lymph nodes; (7) elevated soluble CD25 (sCD25); (8) natural killer (NK) cell activity is low or absent.

### 2.2 Data collection

The clinical data of patients with HNL‐HLH admitted to the First Affiliated Hospital of Nanjing Medical University from October 2010 to June 2015 were collected. All the patients underwent lymph node biopsy and have a pathological diagnosis of HNL. The age, gender, clinical presentation, lymph node signs, laboratory findings and imaging data, and pathological findings of the patients were collected.

## 3 RESULTS

### 3.1 General information

A total of five cases of HNL‐HLH were included in this study, three females and two males, and all patients were adults with a median age of 37 years at diagnosis. None of the patients in this study had a previous history of malignancy or a history of autoimmune disease.

### 3.2 Clinical manifestations

All patients in this study had persistent fevers, with peak temperatures between 38.6°C and 40.8°C. The fevers lasted 17–33 days (medium value: 28 days). All five patients had superficial lymphadenopathy, mainly in the neck, axilla, and groin. Skin rash was observed in only one patient (Table 1). Three patients showed splenomegaly and no patients showed hepatomegaly. No CNS symptoms were present in these five patients.

**Table 1:** General conditions and clinical manifestations.

	Case 1	Case 2	Case 3	Case 4	Case 5
Gender	Female	Male	Male	Female	Female
Age (years)	20	37	40	24	57
History of malignancy	No	No	No	No	No
History of autoimmune disease	No	No	No	No	No
Enlarged lymph nodes	Yes	Yes	Yes	Yes	Yes
Tmax (°C)	40	40.8	39.4	40.2	38.6
Number of days of fever	28	33	25	32	17
Hepatomegaly	No	No	No	No	No
Splenomegaly	No	Yes	Yes	Yes	No
Rash	No	Yes	No	No	No
Emaciation	No	No	No	No	No
Night sweats	No	No	No	No	No
Fatigue	Yes	Yes	No	No	Yes

Abbreviation: Tmax, the maximum value of body temperature.

### 3.3 Laboratory data

Two patients had moderate anemia, and all the five patients had thrombocytopenia. Three patients had leukopenia and neutropenia. Epstein Barr virus and cytomegalovirus DNA were not detected in all peripheral blood samples from the five patients. Case 4 had leukocytosis, elevated erythrocyte sedimentation rate (ESR), and elevated C‐reactive protein (CRP). Elevated liver enzyme levels were found in all five patients. Lactate dehydrogenase (LDH) was significantly elevated in all five patients. Hypertriglyceridemia was present in four patients and only one patient had a fibrinogen level of less than 1.5 g/L. All five patients had hyperferritinemia and the levels of ferritin were all higher than 1000 μg/L (Table 2).

**Table 2:** Baseline laboratory characteristics.

	Case 1	Case 2	Case 3	Case 4	Case 5
HGB (130–175 g/L)	106	89	135	88	131
PLT (125–350 × 10^9^/L)	63	40	67	11	49
WBC (3.5–9.5 × 10^9^/L)	1.3	15	1.78	12.27	1.1
NE (1.8–6.3 × 10^9^/L)	0.5	9.41	0.98	7.18	0.74
β2‐MG (1.0–2.3 mg/L)	NA	NA	NA	2.23	4.38
EBV (<500 copies/mL)	Negative	Negative	Negative	Negative	Negative
CMV (<500 copies/mL)	Negative	Negative	Negative	Negative	Negative
ESR (<21.0 mm/h)	14	12	NA	40	20
CRP (≤6.0 mg/L)	3.36	17.2	3.44	67.4	10.5
ALT (9.0–50.0 U/L)	57.5	175.1	181.8	919.7	278.6
AST (15.0–40.0 U/L)	111.5	398	198.9	1448.1	660.2
LDH (120–250 U/L)	1344	2567	537	4706	986
TG (0.0–2.25 mmol/L)	3.16	4.14	1.2	4.43	3.17
FIB (2.0–4.0 g/L)	1.9	4.66	3.77	5.92	1.47
SF (23.9–336.2 µg/L)	4340	>1500	1450	1159.7	>1500

Abbreviations: ALT, alanine aminotransferase; AST, aspartate aminotransferase; CMV, cytomegalovirus; CRP, C reactive protein; EBV, Epstein Barr virus; ESR, erythrocyte sedimentation rate; FIB, fibrinogen; HGB, hemoglobin; LDH, lactate dehydrogenase; NA, not available; NE, neutrophil; PLT, platelet; SF, serum ferritin; TG, triglyceride; WBC, white blood cell; β2‐MG, β2‐microglobulin.

### 3.4 Diagnosis of HLH

All patients had prolonged and persistent fever, cytopenia involving at least two lineages, and hyperferritinemia. Three patients had splenomegaly, three had elevated sCD25, and hemophagocytosis in the BM was present in four patients. In addition to hemophagocytosis in the BM, hemophagocytes were also observed in the lymph node specimen in case 5. Four patients had hypertriglyceridemia, including one with concomitant hypofibrinogenemia. NK cell activity was not measured in all patients. Ultimately, all patients met at least five of eight HLH‐2004 criteria (Table 3). Additionally, aminotransferase and LDH elevations also supported the diagnosis of HLH.

**Table 3:** HLH‐related test indicators for HLH‐2004 criteria.

	Case 1	Case 2	Case 3	Case 4	Case 5
Body temperature ≥ 38.5°C	Yes	Yes	Yes	Yes	Yes
Splenomegaly	No	Yes	Yes	Yes	No
Cytopenia^a^	Yes	Yes	Yes	Yes	Yes
Hypertriglyceridemia and/or hypofibrinogenemia	Yes	Yes	No	Yes	Yes
Serum ferritin ≥ 500 µg/L	Yes	Yes	Yes	Yes	Yes
Hemophagocytosis in bone marrow	Yes	Yes	Yes	No	Yes
sCD25 pg/mL (≥6400 pg/mL)^b^	NA	15,773	8820	6290	NA
NK cell activity is low or absent	NA	NA	NA	NA	NA
Number of satisfied items	5	7	6	5	5

Abbreviations: HLH, hemophagocytic lymphohistiocytosis; NA, not available; NK, natural killer.

a Cytopenia affecting more than 2 of 3 lineages in the peripheral blood.

^b^ The normal upper limit for sCD25 is 2000 pg/mL and the cutoff for diagnosing HLH is 6400 pg/mL.

### 3.5 Pathological examinations

**Figure 1:**
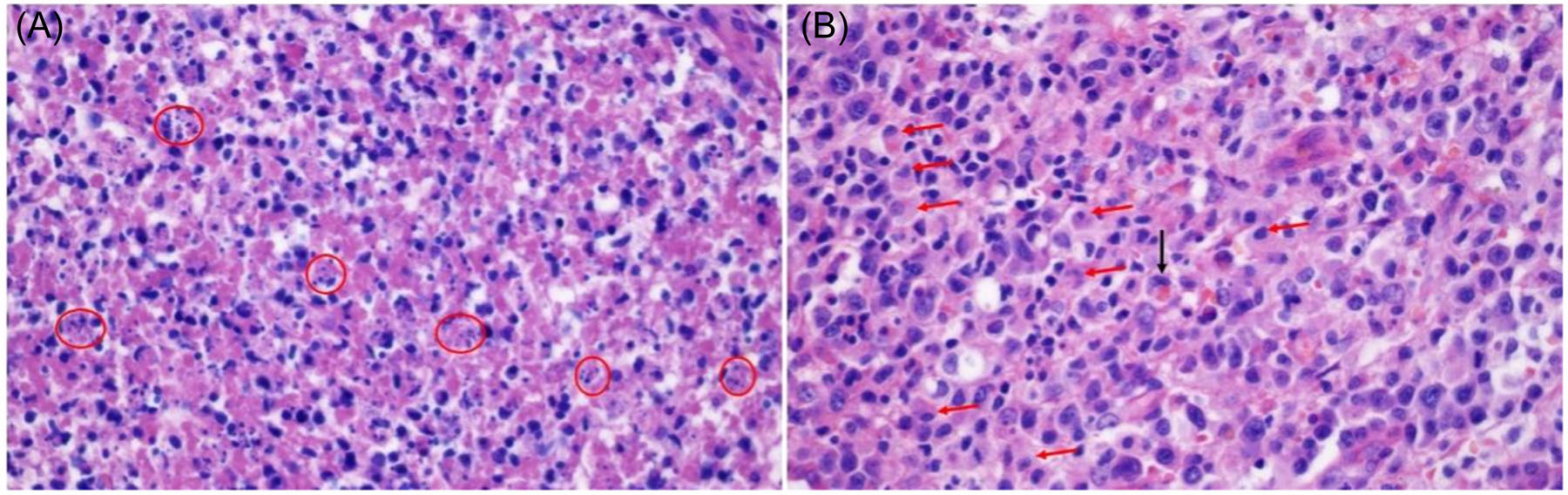
Histological findings of resected lymph node in histiocytic necrotizing lymphadenitis with hemophagocytic lymphohistiocytosis. (A) Extensive karyorrhectic debris was present; the red circles indicated typical karyorrhectic debris. (B) Prominent histiocytic infiltrate with hemophagocytosis was present (case 2); the red arrows indicated histiocytes, and as the black arrow indicated, the histiocyte digested red blood cells.

All five patients in the current underwent a lymph node resection biopsy and all had a pathological diagnosis of HNL. Histologically, paracortical lymph node expansion with patchy, well‐circumscribed areas of necrosis was present in all five cases. Cellular debris and nuclear dust were prominent (Figure 1A). A large accumulation of histiocytes at the edge of necrosis was present in all five cases and neutrophils and eosinophils were absent (Figure 1B). By immunohistochemistry, histiocytes are positive for CD68 and myeloperoxidase. Hemophagocytosis in the lymph node was present in two cases (cases 2 and 5) (Figure 1B).

### 3.6 Image examination

**Figure 2:**
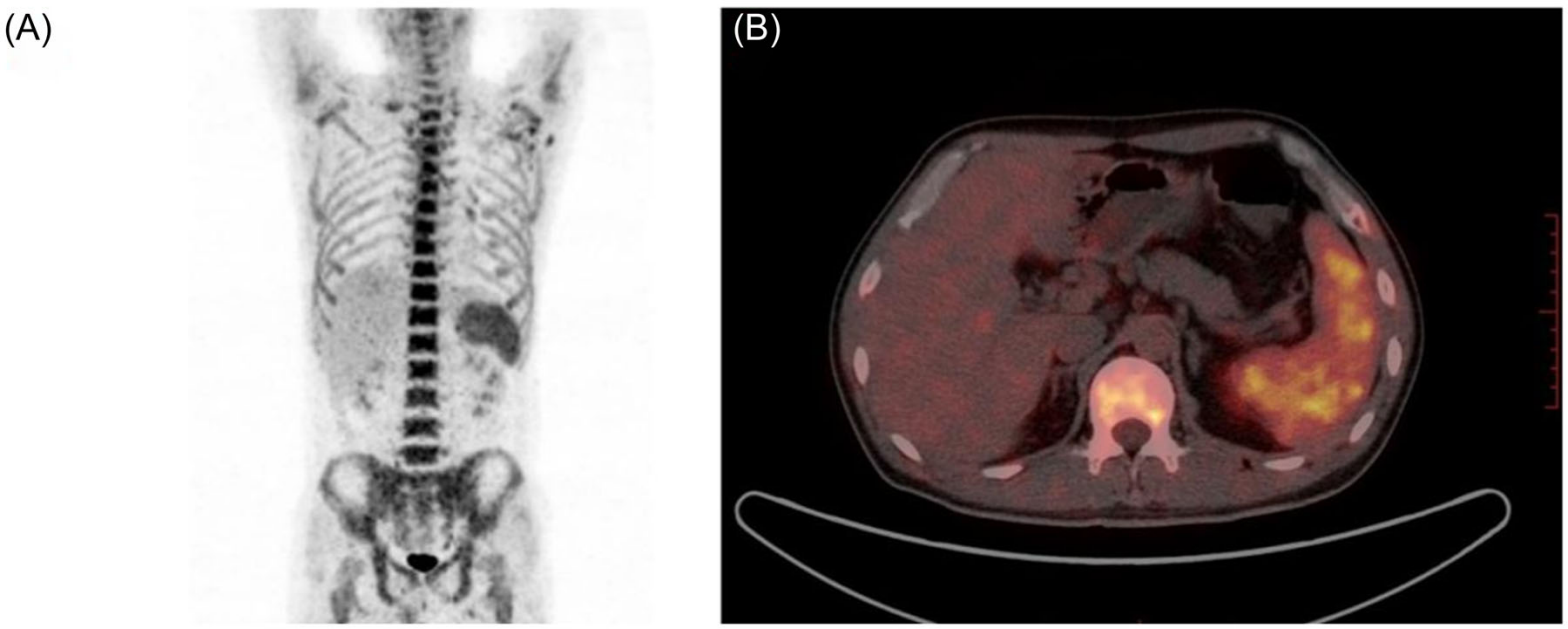
Representative PET/CT images of HNL‐HLH. (A) The lymph nodes, spleen, and bone marrow showed significantly increased FDG uptake (Case 2). (B) The mean standard uptake value of the liver was 1.3 and the spleen had a maximum standard uptake value of 4.5.

18F‐fluorodeoxyglucose positron emission tomography/computed tomography (18F‐FDG PET/CT) was performed on three patients. A representative PET/CT was shown in Figure 2. Enlarged lymph nodes with increased FDG uptake were identified in all three cases. The maximum standard uptake value (SUVmax) of the enlarged lymph nodes in each patient ranged from 2.8 to 4.6. Diffusely increased splenic FDG uptake was present in two cases. And diffusely increased BM uptake was present in all three cases.

### 3.7 Treatment and outcomes

All patients were previously treated with anti‐infective therapy but with poor responses. The symptoms of these five patients all resolved after treatment with glucocorticoid or glucocorticoid‐containing regimens (Table 4). The laboratory parameters also improved (Table 5). Case 5 was discharged in good condition with no complete follow‐up. All other four patients were well followed, and all of them had no recurrence of HNL or HLH. For case 1, 8 months after the diagnosis of HNL‐HLH, the patient was diagnosed with systemic lupus erythematosus (SLE). This case was diagnosed as SLE due to the high titers of antinuclear antibody (1:1000) and anti‐ds‐DNA antibody (541.16 IU/mL), decreased C3 and C4 complement levels, and presence of facial erythema and alopecia. Interestingly, the patient was positive for antinuclear antibody (1:320), anti‐SSA/Ro antibody, and anti‐SSB/La antibody at the diagnosis of HNL‐HLH. Case 3 was diagnosed with SLE 4 months after the diagnosis of HNL‐HLH. He was diagnosed as SLE due to the detection of autoantibodies (antinuclear antibody 1:100, anti‐SSA antibody positive, and anti‐Sm antibody positive), decreased C3 and C4 complement levels, and the presence of recurrent facial erythema and oral ulcer.

**Table 4:** Data on treatment, prognosis, and survival.

	Treatment	Prognosis	Length of follow up (months)
Case 1	Glucocorticoid + IVIG	Survive	110+
Case 2	Glucocorticoid + CTX	Survive	112+
Case 3	Glucocorticoid	Survive	90+
Case 4	Glucocorticoid	Survive	94+
Case 5	Glucocorticoid	Lost to follow‐up	Lost to follow‐up

Abbreviations: CTX, cyclophosphamide; IVIG, intravenous immunogloblin.

**Table 5:** Laboratory results following treatment.

	Case 1	Case 2	Case 3	Case 4	Case 5
HGB (g/L)	142	134	135	82	112
PLT (×10^9^/L)	148	378	61	585	237
WBC (×10^9^/L)	7.8	13.89	2.13	5.02	4.6
NE (×10^9^/L)	4.6	9.11	1.36	2.97	1.88
ESR (mm/h)	9	NA	15	NA	NA
CRP (mg/L)	3.36	8.95	3.44	NA	NA
ALT (U/L)	45.2	57.1	69.3	45.3	127
AST (U/L)	31	23.2	156.1	18.9	98.2
LDH (U/L)	187	264	612	340	482
TG (mmol/L)	1.18	1.15	1.68	1.38	1.43
FIB (g/L)	NA	NA	NA	3.79	3.9
SF (µg/L)	NA	781.7	NA	NA	>1500

Abbreviations: ALT, alanine aminotransferase; AST, aspartate aminotransferase; CRP, C reactive protein; ESR, erythrocyte sedimentation rate; FIB, fibrinogen; HGB, hemoglobin; LDH, lactate dehydrogenase; NA, not available; NE, neutrophil; PLT, platelet; SF, serum ferritin; TG, triglyceride; WBC, white blood cell.

## 4 DISCUSSION

The current study, to the best of our knowledge, described the first case series of adult HNL‐HLH. In this study, we summarized the clinical features and outcomes of five adult patients with HNL‐HLH. All the patients showed a clinical spectrum of HLH that is characterized by overwhelmed inflammation. And the histopathological findings were consistent with that of HNL. All five patients were treated with corticosteroids and have a good prognosis. Two patients were diagnosed with SLE during the follow‐up after the diagnosis of HNL‐HHL, suggesting that this population should be carefully monitored for autoimmune diseases during the routine follow‐up.

HNL is a self‐limiting disease that is more prevalent in Asians than in other populations.^22^ Patients always present with persistent fever and painful lymphadenopathy. Other infrequent symptoms include weight loss, nausea and vomiting, weakness, headache, arthralgia, night sweats, upper respiratory symptoms, and sore throat. Extranodal manifestations including skin lesions have been reported in a small proportion of patients. Laboratory findings include cytopenia, elevated CRP, elevated serum LDH, and elevated aminotransferases. The etiology of HNL remains largely unknown. Pathogens especially viruses could be triggers for HNL. And HNL has also been reported to be associated with autoimmune diseases including SLE, Sjögren syndrome, Still disease, and others, suggesting autoimmune disease could lead to the development of HNL in some patients. In a study involving 91 patients HNL, 11 patients were diagnosed with SLE, suggestion the association between HNL and SLE.^23^ HNL could resolve within a few months without any specific treatments. Patients with an aggressive clinical course could be treated with steroids.

A cohort of 13 pediatric cases of HNL‐HLH has been described.^5^ According to this study, skin rashes, hepatosplenomegaly, and CNS symptoms are common in pediatric cases. And common laboratory abnormalities include cytopenia, hypertriglyceridemia, and hyperferritinemia. Hemophagocytosis in BM or lymph nodes is common, suggesting that hemophagocytosis is a pathological feature for patients with HNL‐HLH. Most patients respond well to corticosteroids and have a good prognosis. Two cases developed autoimmune diseases during the follow‐up after the diagnosis of HLH.

As compared to HNL patients without HLH, patients with HNL‐HLH showed prolonged fever and laboratory findings that suggest a hyperinflammatory state. In addition to markers diagnostic of HLH, HNL‐HLH patients show remarkably higher levels of LDH as compared with classical HNL patients.^23^ 18F‐FDG PET/CT of three adult patients with HNL‐HLH showed enlarged lymph nodes with moderately increased FDG uptake, which is consistent with findings from patients with HNL.^24^

Only a proportion of patients have a hypermetabolic spleen on the 18F‐FDG PET/CT.^24^ According to the study by Seong et al., Splenic uptake on FDG PET/CT correlates with Kikuchi‐Fujimoto disease severity.^25^ In our study, significantly increased FDG uptake of the spleen was present in two cases, suggesting that hypermetabolic spleen may be a characteristic radiological feature for patients with HNL‐HLH. Adult patients with HNL‐HLH may display distinct clinical features from pediatric patients. For example, compared with pediatric cases, skin rash was less frequent in adult cases and no patients showed CNS manifestations.^5^

The link between HNL and HLH remains to be determined. Caocci et al. reported a case of HNL‐HLH following the BNT162b2 mRNA COVID‐19 vaccination, suggesting that HNL and HLH may share common underlying causes.^15^ For example, viruses could contribute to the development of HNL as well as HLH. And Notaro et al report a patient with HNL associated with recurrent subcutaneous panniculitis‐like T‐cell lymphoma (SPTL). This patient was also diagnosed with HLH. As SPTL could be complicated with HLH, in this case, SPTL could contribute to the development of HLH as well as HNL.^21^ Interestingly, patients with SPTL frequently developed HLH and germline HAVCR2 mutations contribute to SPTL and HLH at the same time in some cases.^26^, ^27^ HNL could also directly contribute to the pathogenesis of HLH. Activated T cells are abundant in HNL and excessively activated T cells produce uncontrolled cytokines, thereby leading to the emergence of HLH.

Our study demonstrated that adult patients with HNL‐HLH showed distinct clinical, laboratory, imaging, and pathological features. And the prognosis is good and patients could be managed with steroids and supportive care. Regular follow‐up is mandatory as a proportion of patients could develop autoimmune diseases after the diagnosis. Further studies are needed to elaborate on the mechanisms underlying the pathogenesis of HNL‐HLH.

## AUTHOR CONTRIBUTIONS

**Qingqing Chen**: Conceptualization; data curation; formal analysis; methodology; visualization; writing—original draft. **Jing Zhang**: Conceptualization; data curation; formal analysis; methodology; visualization; writing—original draft. **Huijun Huang**: Data curation; formal analysis; visualization. **Tonglu Qiu**: Data curation; formal analysis; visualization. **Ze Jin**: Data curation; formal analysis; visualization. **Yu Shi**: Data curation; Formal analysis; Visualization. **Huayuan Zhu**: Conceptualization; supervision; writing—review and editing. **Lei Fan**: Conceptualization; supervision; writing—review and editing. **Jianyong Li**: Conceptualization; funding acquisition; methodology; supervision; writing—review and editing. **Wenyu Shi**: Conceptualization; funding acquisition; methodology; supervision; writing—review and editing. **Yi Miao**: Conceptualization; funding acquisition; methodology; supervision; writing—review and editing.

## CONFLICT OF INTEREST STATEMENT

The authors declare no conflict of interest.

## ETHICS STATEMENT

The study was approved by the Ethics Committee of Jiangsu Province Hospital (2023‐SR‐572). All patients signed informed consent forms.

## Data Availability

The data that support the findings of this study are available on request from the corresponding author.
